# Comparative Analysis of Large Language Models in Chinese Medical Named Entity Recognition

**DOI:** 10.3390/bioengineering11100982

**Published:** 2024-09-29

**Authors:** Zhichao Zhu, Qing Zhao, Jianjiang Li, Yanhu Ge, Xingjian Ding, Tao Gu, Jingchen Zou, Sirui Lv, Sheng Wang, Ji-Jiang Yang

**Affiliations:** 1 College of Computer Science, Beijing University of Technology, Beijing 100124, China; Zhuzc@emails.bjut.edu.cn (Z.Z.); zhaoqing@bjut.edu.cn (Q.Z.); lijianqiang@bjut.edu.cn (J.L.); dxj@bjut.edu.cn (X.D.); 17367571015@163.com (T.G.); zoujc2001@163.com (J.Z.); lsr191203@163.com (S.L.); 2 Department of Anesthesiology, Beijing Anzhen Hospital, Capital Medical University, Beijing 100013, China; yanhu_ge@hotmail.com; 3 Department of Automation, Tsinghua University, Beijing 100084, China

**Keywords:** large language model, biomedical named entity recognition, electronic medical record

## Abstract

The emergence of large language models (LLMs) has provided robust support for application tasks across various domains, such as name entity recognition (NER) in the general domain. However, due to the particularity of the medical domain, the research on understanding and improving the effectiveness of LLMs on biomedical named entity recognition (BNER) tasks remains relatively limited, especially in the context of Chinese text. In this study, we extensively evaluate several typical LLMs, including ChatGLM2-6B, GLM-130B, GPT-3.5, and GPT-4, on the Chinese BNER task by leveraging a real-world Chinese electronic medical record (EMR) dataset and a public dataset. The experimental results demonstrate the promising yet limited performance of LLMs with zero-shot and few-shot prompt designs for Chinese BNER tasks. More importantly, instruction fine-tuning significantly enhances the performance of LLMs. The fine-tuned offline ChatGLM2-6B surpassed the performance of the task-specific model BiLSTM+CRF (BC) on the real-world dataset. The best fine-tuned model, GPT-3.5, outperforms all other LLMs on the publicly available CCKS2017 dataset, even surpassing half of the baselines; however, it still remains challenging for it to surpass the state-of-the-art task-specific models, i.e., Dictionary-guided Attention Network (DGAN). To our knowledge, this study is the first attempt to evaluate the performance of LLMs on Chinese BNER tasks, which emphasizes the prospective and transformative implications of utilizing LLMs on Chinese BNER tasks. Furthermore, we summarize our findings into a set of actionable guidelines for future researchers on how to effectively leverage LLMs to become experts in specific tasks.

## 1. Introduction

The widespread deployment of electronic medical record (EMR) systems has made the richness of various clinical data resources increasingly prominent [1,2], such as laboratory test results, medication treatments, and diagnostic information. These data have emerged as a valuable repository for large-scale clinical data analysis [3,4,5,6,7,8]. However, the narrative nature of EMRs somewhat restricts the potential for data reuse. Against this background, as a profound application and extension of named entity recognition (NER) technology in the medical field, biomedical named entity recognition (BNER) can automate the identification of named entities within unstructured EMR text and categorize them into predefined classes such as diseases, symptoms, tests, and treatments [9]. By precisely and comprehensively extracting relevant information about target patient populations, BNER technology provides a solid foundation for the construction of structured clinical information systems, clinical decision support systems, and medical knowledge graphs [10,11,12,13,14], thereby offering robust support for the practice and development of evidence-based medicine [1,2,15].

Recently, with the rise of foundational models [16,17], a new paradigm utilizing deep learning models has been introduced in natural language processing (NLP). The paradigm depends on the emerging abilities of large language models (LLMs) [18] to handle more complex tasks through scaling. Unlike training specialized models for specific issues, a large general foundational model is trained once to acquire general knowledge, which can then be leveraged (via prompting) for numerous other subsequent tasks. This paradigm is introduced by language models as few-shot learners [19], and gains widespread recognition with the launch of groundbreaking ChatGPT models, including GPT-3.5 [20] and GPT-4 [21], which incorporated techniques like reinforcement learning with human feedback (RLHF) [20]. The performance of LLMs is explored in various domains, including the NER task [22]. However, these explorations of NER primarily focus on English and general domains. There is still limited validation and in-depth exploration regarding how to understand, assess, and enhance LLM’s capabilities in BNER tasks, particularly when applied to Chinese clinical records. Therefore, the objective of this study is to expand the evaluation of LLMs by validating their capabilities on Chinese BNER tasks, thereby exploring improved prompts, larger-scale assessments, and a broader range of NER tasks.

We conducted a series of Chinese BNER experiments on multiple LLMs, evaluating them leveraging a real-world EMR dataset and a publicly available dataset with high-quality entity annotations. Our experiments consisted of three stages: (1) zero-shot prompting, where we input various prompts related to Chinese BNER, (2) few-shot prompting, where we inserted examples into prompt inputs, and (3) instruction fine-tuning, where we fine-tuned LLMs on the datasets.

The experimental results indicate that zero-shot prompting received a promising yet limited performance on Chinese BNER tasks. The ChatGPT models, particularly GPT-4, showed relatively better performance due to it having a larger number of parameters. However, they still have a significant gap with the supervised learning method. Providing a few shots in prompts can improve the performance of LLMs to some extent, but the improvement is limited. Impressively, we found instruction fine-tuning significantly enhances the performance of LLMs on Chinese BNER tasks. The fine-tuned offline ChatGLM2-6B surpasses the original ChatGPT models (GPT-3.5 and GPT-4) in terms of recognition accuracy. It is worth noting that the parameter size of ChatGLM2-6B is only 6 billion, while ChatGPT has parameters exceeding hundreds of billions. Furthermore, the fine-tuning results of ChatGLM2-6B on the pregnancy complicated by heart disease (PCHD) and CCKS2017 datasets even outperformed the supervised learning model BiLSTM+CRF (BC). GPT-3.5, after fine-tuning on the CCKS2017 dataset, achieves the best performance among all LLMs, surpassing even half of the baseline models. However, unfortunately, these fine-tuned LLMs still fall short of surpassing the state-of-the-art Dictionary-guided Attention Aetwork (DGAN) [13]. The primary reason is that DGAN benefits from a meticulously designed model architecture and knowledge guidance tailored for specific domain tasks, enabling it to better understand and capture finer differences and semantic features. In contrast, LLMs are primarily trained on large-scale unlabeled text data in general domains to provide a broad understanding of various tasks. Fine-tuning can only add a portion of domain-specific knowledge, but falls short of achieving the best possible performance. The contributions of this study can be summarized as follows:

(1) Executing a wide evaluation of the performance of LLMs on Chinese BNER tasks. To our knowledge, this is the first exploration of LLMs on Chinese BNER tasks.

(2) The BNER task on private EMRs is implemented leveraging the offline ChatGLM2-6B, providing a reference for the performance of LLMs on real-world data.

(3) The effects of zero-shot, few-shot, and instruction fine-tuning on the performance of LLMs are comprehensively measured leveraging the real-world dataset and the public dataset. The experimental results indicate that fine-tuning on datasets can significantly improve the capability of LLM on Chinese BNER tasks.

(4) We provide a few guidelines for future researchers on turning LLMs into domain-specific experts.

The paper is organized as follows: we discuss related work in the next section. The method is presented in Section 3. Section 4 outlines the experiment setup. The experimental results charted out in Section 5. Finally, Section 6 and Section 7 provide concluding remarks.

## 2. Related Work

### 2.1. Biomedical Named Entity Recognition

Recently, studies on BNER have primarily focused on deep neural network methods. Chokwijitkul et al. [23] investigated the abilities of Convolutional Neural Network (CNN), Recurrent Neural Network (RNN), Long Short-Term Memory (LSTM), Bidirectional Long Short-Term Memory (BILSTM), and Gated Recurrent Unit (GRU) models in recognizing cardiovascular risk factor entities in clinical data. Among them, BILSTM achieved the best performance. Differing from the single-model evaluation conducted by Chokwijitkul et al. [23], many other approaches extend the model by incorporating multiple network layers, such as character embedding layers, BILSTM, and Conditional Random Field (CRF). The experimental results of Xu et al. [24] and Unanue et al. [25] indicate that the construction pattern of multi-model ensembling can effectively enhance the performance of the models. This is as expected, since different network layers can extract distinct semantic features, providing the model with additional useful information and effectively improving its discriminative capability. Furthermore, some approaches enhance the model’s capability to focus on crucial information by introducing attention mechanisms, thus further enhancing the performance of the model in NER tasks. For instance, Li et al. [26] constructed a BILSTM-CRF model and employed a bidirectional maximum matching method to extract entities from EMRs, further capturing important semantic information through integrated the attention mechanism. In addition, incorporating domain knowledge can greatly enhance the performance of models in BNER tasks. Xu et al. [27] developed a Dic-Att-BILSTM-CRF model, which augmented the model with medical guidance by integrating a medical dictionary. In our previous work [28], we developed a DGAN model that achieved the best performance among all models by integrating a medical dictionary to provide knowledge-guided weight allocation for the attention mechanism.

### 2.2. Large Language Models and In-Context Learning

LLMs [19,29,30,31,32] have achieved remarkable performance improvements in various natural language processing tasks [33,34,35,36,37]. The strategies for applying LLMs to downstream tasks can be categorized into two types: fine-tuning and in-context learning. The fine-tuning strategy initializes the pretrained model and runs additional epochs on the supervised dataset [38,39,40,41].

Unlike the fine-tuning strategy, LLMs like ChatGPT (GPT-3.5 and GPT-4) have introduced a novel paradigm called in-context few-shot learning. The paradigm does not require parameter updates, and can receive excellent results with just a few examples about the downstream task. Since the strong relation between the effectiveness of in-context learning and the selection of demonstration examples, recent studies explore multiple effective example selection methods, e.g., similarity-based retrieval methods [42,43], validation score-based selection methods [44], and gradient-based methods [45]. The experimental results of these methods have demonstrated that proper example selection can effectively enhance the performance of large-scale language models.

## 3. Methodology

In this section, we introduce our design with LLMs on the setup if Chinese BNER task, including zero-shot prompting, few-shot prompting, and instruction fine-tuning.

### 3.1. Zero-Shot Prompting

The language understanding and reasoning capability of LLMs have enabled a wide range of applications without the need for any domain-specific data, but only providing appropriate prompts [37,46]. Therefore, we start with prompt design for Chinese BNER tasks in a zero-shot setting. Zero-shot prompting [47] is a method that leverages pre-trained large language models (LLMs) to solve tasks directly without any specific task training samples. This approach relies on the general knowledge acquired by the model during pre-training on vast amounts of data and uses natural language prompts to guide the model in accomplishing particular tasks.

The goal of prompt design is to empower a pre-trained general-purpose LLM to achieve good performance. In this study, we propose a general zero-shot prompt template (${\mathrm{Prompt}}_{Zero-shot}$) that consists of four parts:

(1) $$\begin{array}{c} Prompt_{Zero-shot}=Input\_text+S\_text+Q\_text+Output\_Control, \end{array}$$

where *Input_text* is the input EMR text. *S_text* provides specifications for a Chinese BNER task. *Q_text* is the question for LLMs to answer. *Output_Control* controls the output of LLMs (e.g., we require LLMs to label the biomedical entities with char-level). We present multiple design strategies for *S_text*, as shown in Table 1: (1) basic, leaving it as blank; (2) context enhancement, providing more context information about the *Input_text*; (3) role-playing, letting the model act as a medical expert, positioning LLM within a specific domain to highlight its expertise in that field, which is an effective method to enhance LLM’s performance in professional tasks [48]; and (4) context and role-playing, combining strategies (2) and (3), letting the model act as a medical expert under the background of more context information. As for *Q_text*, we focus on the targets of Chinese BNER: (1) recognizing certain Chinese biomedical entities, e.g., “呼吸困难” (dyspnea), “超声心动图” (echocardiogram), “妊娠合并心脏病” (pregnancy complicated by heart disease), etc.; (2) predicting the types of certain entities, e.g., symptom, test, disease, etc. We tailor the question description for the two targets, as shown in the bottom part of Table 1.

### 3.2. Few-Shot Prompting

To provide more domain-specific information, we also explore the effect of few-shot prompting with LLMs. Few-shot prompting [19] involves providing a small number of task examples (typically one to a few) within the prompt, enabling the model to better grasp the expected output format for the task. This method harnesses the language model’s ability to understand context and, without specific fine-tuning, teaches the model on how to complete the task by embedding these limited examples within the prompt. It is important to note that these few examples serve only as a prompt, and the model’s parameters remain frozen. Additionally, in this study, we also evaluate this strategy by supplementing additional randomly sampled [*Sample ${\mathrm{Prompt}}_{Few-shot}$-label*] pairs. The design of the few-shot prompt (${\mathrm{Prompt}}_{Few-shot}$) is as shown in (2),

(2) $$\begin{array}{c} Prompt_{Few-shot}={\left[SamplePrompt_{Zero-shot}-label\right]}_{M}+Prompt_{Zero-shot}, \end{array}$$

where *M* is the number of the prompt-label pairs and is capped by the input length limit of a model. Meanwhile, both the *Sample* ${\mathrm{Prompt}}_{Zero-shot}$ and ${\mathrm{Prompt}}_{Zero-shot}$ follow (1) and employ the same design of *S_text* and *Q_text* to ensure consistency.

### 3.3. Instruction Fine-Tuning

Instruction fine-tuning [37] refers to the process of fine-tuning a model after pre-training with additional instruction data, enabling it to better understand and execute given commands. This fine-tuning typically involves a collection of specific instruction tasks, and is conducted through multi-task learning, where the model not only can learn the content of the tasks, but also how to perform tasks based on instructions.

During the process of fine-tuning, we follow two steps.

Step 1: fine-tune with [${\mathrm{Prompt}}_{Zero-shot-train}$− *label*]$\sum_{i=1}^{l}N_{D_{i=train}}$

Step 2: Test with [${\mathrm{Prompt}}_{Zero-shot-train}$]$\sum_{i=1}^{l}N_{D_{i=test}}$, where *D* is the total number of the dataset. $N_{D_{i=train}}$ and $N_{D_{i=test}}$ are the number of the train dataset and test dataset, respectively. *I* denotes the datasets leveraged for fine-tuning. *i* denotes the index in datasets. ${\mathrm{Prompt}}_{Zero-shot-train}$ and ${\mathrm{Prompt}}_{Zero-shot-test}$ follow (1). Similarly to the setup of few-shot in (2), we employ the same design of *S_text* and *Q_text* to ensure consistency.

## 4. Experiments Set

### 4.1. Dataset

The experiments are based on two specialized medical datasets obtained from a cooperative hospital, manually labeled by the guidance of professional experts and teams, namely: PCHD: A real-world Chinese dataset about pregnancy complicated by heart disease (PCHD), which contains 138 EMRs and labeled with five types of entities, i.e., “症状” (symptom), “检查” (test), “检查结果” (test result), “疾病” (disease), and “治疗” (treatment). After data processing, a total of 8000 sentences are available for experimentation, with 7000 sentences utilized as the training set and 1000 sentences used as the test set.

CCKS2017: A public Chinese dataset provided by Beijing Jimuyun Health Technology Co. which contains 800 records and annotated with five types of entities, including “身体部位” (body), “症状” (symptom), “检查” (check), “疾病” (disesase), and “治疗” (treatment). We segment these annotated records into sentences and divide them into training and testing sets with a ratio of 7:1. The dataset is available at: https://www.heywhale.com/mw/dataset/648058405742d97f8f6beca0/file, accessed on 7 June 2023.

### 4.2. Evaluation Metrics

We leverage precision *P*, recall *R*, and *F*1 value to evaluate the performance of LLMs on Chinese BNER tasks. The specific calculation process of *P*, recall *R*, and *F*1 are shown in Equations (3)–(5):

(3) P=TP/(TP+FP),

(4) R=TP/(TP+FN),

(5) F1=2×P×R/(P+R),

where *TP*, recall *FP*, and *FN* denote the number of correctly recognized biomedical entities, the number of unrelated biomedical entities recognized, and the number of unrecognized biomedical entities, respectively.

### 4.3. Models

We conducted experiments on representative baseline models and multiple LLMs with different sizes. The baselines include BERT and DGAN, while the LLMs include ChatGLM2-6B, GLM-130B, GPT-3.5, and GPT-4. The detailed descriptions of these models are as follows:

BC [24]: this model uses word embeddings and the BILSTM-CRF model for NER.

BERT [49]: A classic language representation model designed to pretrain deep bidirectional representations by jointly considering left and right context in all layers. It can be fine-tuned with just one additional output layer to create models for various downstream tasks.

- Source code: https://github.com/ymcui/Chinese-BERT-wwm, accessed on 2 November 2021.

BBC [50]: this model utilizes BERT to generate the character embeddings and uses the BiLSTM-CRF model for NER.

RSBGC [51]: this model adopts RoBERTa to generate word embeddings, and leverages the Stacked BiGRU-CRF framework for NER.

FBBCE [52]: this model utilizes the domain-specific medical knowledge and BERT to generate character embeddings, and then the BILSTM-CRF model is used to recognize entities.

DABLC [27]: this model extracts concepts from the external dictionary to improve the standard attention mechanism for BNER.

DGAN [28]: A domain-specific neural network model that focuses on utilizing medical dictionary knowledge to enhance the attention mechanism’s capability to focus on the overall medical entity. It has demonstrated outstanding performance in both private and public data for BNER tasks.

ChatGLM2-6B: ChatGLM-6B is an open-source language model that supports both Chinese and English languages, with 6.2 billion parameters. The model can be fine-tuned by various techniques such as supervised learning and human feedback. Further, it can run on consumer grade graphics cards with only 6GB of memory since the quantization technology.

- Source code: https://github.com/thudm/chatglm2-6b, accessed on 25 June 2023.
- Unofficial demo: https://huggingface.co/spaces/mikeee/chatglm2-6b-4bit, accessed on 25 June 2023.

GLM-130B: GLM-130B has 130 billion parameters. Similarly to ChatGLM2-6B, GLM-130B also supports bilingual Chinese and English. The objective of GLM-130B is to provide an open-source alternative solution comparable to the scale of GPT-3.

- Source code: https://github.com/THUDM/GLM-130B, accessed on 31 July 2023.
- Official online demo: https://chatglm.cn/detail, accessed on 31 July 2023.

GPT-3.5: GPT-3.5 is closed-source and available by API provided by OpenAI. The model has 175 billion parameters, and has been demonstrated to possess excellent performance in multiple NLP tasks.

- Web application: https://chat.openai.com/, accessed on 30 November 2022.

GPT-4: GPT-4 is the most powerful model of OpenAI. The model stronger than GPT-3.5 in quantitative questions (math and physics), creative writing, and many other challenging tasks, which exhibits human-level performance across a range of professional and academic benchmarks.

- Web application: https://chat.openai.com/, accessed on 14 March 2023.

### 4.4. Experimental Environment

For the ChatGLM2-6B model, including its zero-shot, few-shot prompting, and instruction fine-tuning, we conducted the experiments by locally configuring and loading the model. The hardware configuration is detailed in Table 2.

As for the larger-scale models GLM-130B, GPT-3.5, and GPT-4, due to limited local hardware resources (for GLM-130B) and closed-source restrictions (for GPT-3.5 and GPT-4), we carry out their zero-shot prompting, few-shot prompting, and instruction fine-tuning by leveraging the official APIs for remote interaction to accomplish the tasks.

## 5. Experiment Results

We summarize the experiment results with zero-shot prompting, few-shot prompting, and instruction fine-tuning, as shown in Table 3 and Table 4. Note that due to privacy concerns, the real-world PCHD dataset is exclusively utilized for fine-tuning and experiments on the offline ChatGLM2-6B model.

### 5.1. Zero-Shot Prompting

The performance of the zero-shot prompting is summarized in the upper section of Table 3 and Table 4. The results of Basic indicate that a larger model performs better on Chinese BNER tasks without any prompting information, in particular GPT-4’s performance on the CCKS2017 dataset improves by 0.109 on the basis of ChatGLM2-6B. This demonstrates that LLMs are promising for Chinese BNER tasks. However, there is still a significant gap compared to the task-specific baselines. Moreover, in Section 3.1, we present context enhancement, role-playing, and the combination of context enhancement and role-playing for zero-shot prompt design to supplement more useful information to improve the performance of LLMs. Table 5 shows a zoom-in summary of the enhancement strategies of zero-shot prompting in Table 3 and Table 4. The ↑ and ↓ in Table 5 denote increased accuracy and decreased accuracy, respectively. Note that this table is computed according to the Basic strategy of zero-shot.

For different LLMs, all three strategies improve their performance on Chinese BNER tasks. While the role-playing strategy provides the worst improvement effect, it still plays a positive utility. Compared to the Basic strategy, the role-playing strategy yields an approximate improvement of 0.096 on the PCHD dataset and an approximate improvement of 0.011 to 0.062 on the CCKS2017 dataset. The context enhancement strategy outperforms the role-playing strategy, with an approximate improvement of 0.152 and 0.061 to 0.126 on the PCHD and CCKS2017 dataset compared to the Basic strategy. The combination of the two strategies (context enhancement and role-playing) receives the best effects, with the approximately 0.147 and 0.076 to 0.155 performance improvement on the PCHD and CCKS2017 datasets, respectively. In particular, the combination of the two strategies receives more significant improvement in the performance of larger scale GLM-130B, GPT-3.5, and GPT-4, which indicates that the LLMs with larger scale parameters can better utilize the information embedded in prompts. However, the improvement of the LLMs through the construction of enhancement strategies remains severely limited, with a significant gap compared to task-specific baseline models (BiLSTM+CRF, BERT and DGAN). This is because baseline models are designed and trained more meticulously for specific tasks, enabling them to more comprehensively explore and exploit contextual semantic information. In contrast, enhancement strategies can only provide coarse background knowledge to LLMs, which cannot compare to task-specific learning.

Overall, we can summarize the key points below.

(1) LLMs exhibit promising performance on Chinese BNER tasks with zero-shot prompting, but their capabilities are still not comparable to the task-specific baseline model.

(2) Designing enhancement strategies of prompts are generally effective.

(3) LLMs with larger scale parameters can better leverage the information embedded in prompts.

### 5.2. Few-Shot Prompting

We explore the effectiveness of few-shot prompting in this section. The LLM’s best performance with few-shot prompting are summarized in the middle part of Table 3 and Table 4. Note that due to the different effects of prompt design strategies shown in Table 1, in this section, we only leverage the prompts which receive the best performance in the setting of zero-shot prompting. We conduct five repetitions of the experiment for the task, randomly allocating few-shot samples for each run. Table 6 is a zoom-in summary of few-shot prompting in Table 3 and Table 6. In Table 6, ↑ represents increased accuracy, and ↓ denotes decreased accuracy. Note that this Table is computed based on the “both” strategy of zero-shot.

It is observed from Table 3, Table 4 and Table 6 that although LLMs with few-shot prompting still underperform the task-specific baseline model, providing examples of the Chinese BNER task can better improve the performance of LLMs compared to zero-shot prompting. Interestingly, the few-shot prompting is more effective for ChatGLM2-6B with smaller parameter sizes. For the CCKS2017 dataset, under 5-shot and 10-shot settings, we observed improvements of 0.036 and 0.040 in the F1 score for ChatGLM, while GLM, GPT-3.5, and GPT-4 showed increases of 0.011 and 0.034, 0.021, and 0.027, as well as 0.030 and 0.038, respectively. Therefore, ChatGLM outperforms GLM-130B, GPT-3.5, and GPT-4 in terms of performance improvement. This could be attributed to the fact that smaller models like ChatGLM2-6B are more capable of quickly adapting to new tasks, even with just a few examples. While the large models like GLM-130B, GPT-3.5, and GPT-4 have a lot things “in memory”, and find it challenging to learn quickly from examples. Meanwhile, LLMs exhibit a more significant improvement under the 10-shot setting compared to 5-shot. This is because the greater number of shots enables the LLM to observe a broader diversity of training samples, thereby acquiring richer information to improve the performance.

The above results highlight the key finding of this experiment:

(1) Few-shot prompting can boost the performance of LLM in BNER tasks.

(2) Few-shot prompting may be more effective for LLMs with smaller parameter sizes.

(3) Having more shots means that the LLM can observe a greater number of training samples, thus acquiring richer information. This aids the model in better understanding tasks, categories, or patterns, ultimately enhancing its performance.

### 5.3. Instruction Fine-Tuning

In this section, we then investigate the effectiveness of instruction fine-tuning. We only perform instruction fine-tuning on ChatGLM2-6B and GPT-3.5 because of the high costs. Similar as the selection of the best prompts in Section Section 5.3, we also adopt the best prompts in the process of instruction fine-tuning to ensure consistency. To comprehensively assess the impact of instruction fine-tuning on the performance of the LLM, we conducted a thorough analysis from multiple perspectives. The details are introduced below.

#### 5.3.1. The Comparative Results with Existing State-of-the-Art Methods

By analyzing the comparative experimental results from Table 3 and Table 4, several crucial conclusions can be observed.

(1) Instruction fine-tuning is more effective for the smaller-sized ChatGLM2-6B model. The amplification summary of LLMs’ performance improvement is illustrated in Table 7 (↑ represents increased accuracy, and ↓ denotes decreased accuracy. Note that Table 7 is computed based on the “both” strategy of zero-shot). This observation is akin to few-shot methods (Section 5.3), with the primary reason likely attributed to the ability of smaller-sized LLMs to adapt to new tasks more rapidly through fine-tuning. In contrast, the larger parameter scale of GPT-3.5 possesses an abundance of “memory” data, making it challenging to achieve rapid learning from new instances.

(2) Fine-tuning models with larger size parameters remains the primary choice for achieving high accuracy. While the performance improvement after fine-tuning is more pronounced in smaller-sized LLMs, their achievable accuracy is significantly lower compared to larger LLMs, as illustrated in Table 8. After fine-tuning, ${\mathrm{GPT-3.5}}_{CCKS}$ achieved the highest accuracy among all fine-tuned models, with improvements in F1 values compared to ${\mathrm{ChatGLM2-6B}}_{PCHD}$, ${\mathrm{ChatGLM2-6B}}_{CCKS}$, and ${\mathrm{ChatGLM2-6B}}_{PCHD\&CCKS}$ of 0.212, 0.061, and 0.048, respectively. This is primarily due to the larger size parameter of LLMs, which endows them with more robust representational and learning capabilities. Larger-size models can learn richer and more abstract feature representations, enabling the model to better comprehend the underlying patterns and relationships in the data, thereby achieving higher accuracy.

(3) Even after fine-tuning, LLMs still struggle to surpass the optimal task-specific baseline models. As indicated in Table 8, after fine-tuning, the three fine-tuned ChatGLM2-6B models only exhibit a slight advantage over the BC model. The primary reason lies in the fact that the BC model is built upon a simple model structure of BILSTM+CRF, which significantly lags behind in terms of parameter scale, feature representation, and feature learning compared to the fine-tuned ChatGLM2-6B. In contrast, the other six baseline models have surpassed the performance of the fine-tuned ChatGLM2-6B by integrating BERT models or incorporating domain-specific knowledge. These models have undergone extensive enhancements and more thoughtful designs in terms of parameter size, feature representation, and feature learning. Furthermore, our best fine-tuned model, ${\mathrm{GPT-3.5}}_{CCKS}$, outperformed more than half of the baseline models (i.e., BC, BERT, BBC and RSBGC) but still fell short of the optimal performance. The main reasons can be summarized in the following aspects: (1) Architectural differences: The network structures of smaller models like FBBCE, DABLC, and DGAN, which are meticulously designed for specific domain BNER tasks. In particular, the CRF layers they employ are highly adept at handling and capturing the relationships between named entities, thus inferring the globally optimal solution. In contrast, the base model of ${\mathrm{GPT-3.5}}_{CCKS}$, GPT-3.5, is not specifically designed for BNER tasks. Its complex network structure makes it difficult to focus on the features relevant to specific BNER tasks. Additionally, FBBCE, DABLC, and DGAN have a smaller number of parameters with a more compact network structure, making them easier to converge on the limited datasets. Unlike them, ${\mathrm{GPT-3.5}}_{CCKS}$ has a vast number of parameters, and while it can be adapted to NER tasks through fine-tuning, this typically requires a larger annotated dataset, which is not easy to obtain. And (2) domain-specific knowledge: Smaller models like FBBCE, DABLC, and DGAN integrate domain-specific knowledge from fine-tuned BERT and external medical dictionaries. This knowledge is often rich and extensive, and by designing more refined loss functions to make full use of it, the models can learn more valuable information and unique insights, thereby effectively enhancing accuracy. In contrast, although ${\mathrm{GPT-3.5}}_{CCKS}$ incorporates some domain-specific knowledge after fine-tuning, this knowledge primarily comes from the dataset and lacks in-depth domain expertise. Moreover, the loss functions used during fine-tuning may not align effectively with the GPT-3.5-specific learning objectives for the given task, thus limiting the potential adaptability of GPT-3.5. In conclusion, although ${\mathrm{GPT-3.5}}_{CCKS}$ has demonstrated commendable performance, it still falls short of the optimal level.

#### 5.3.2. The Effects of Data Content

In general, that the LLMs that are fine-tuned and tested on the same datasets can lead to good performance is not surprising, but this also raises a question: do the fine-tuned LLMs generalize across other datasets? To verify this, we adopt two different strategies to fine-tune LLMs: (1) utilizing a single dataset (PCHD or CCKS) to fine-tune LLMs; and (2) fine-tuning LLMs using a mixed dataset (PCHD and CCKS). To ensure the reliability of the experiments, the scale of the mixed dataset is 3000, where the probability of PCHD and CCKS in the mixed dataset is 1:1.

The experimental results are shown in the last part of Table 2 and Table 3. From the perspective of generalization capability, LLMs fine-tuned on the single dataset (PCHD or CCKS) is significantly lower than that of mixed data (PCHD and CCKS), which matches the expectation. This is because the significant content changes contained in the mixed data can provide more differential information to the model, thus effectively improving the discriminatory capability of LLMs.

#### 5.3.3. The Effects of Data Scale

The scale of data are generally proportional to the performance of the model. However, the cost of obtaining the large-scale high-quality annotated data and fine-tuning LLMs is prohibitive. Therefore, we hope to explore the relation between the performance of fine-tuned LLMs and the scale of data to save costs in resource-limited settings.

**Figure 1:**
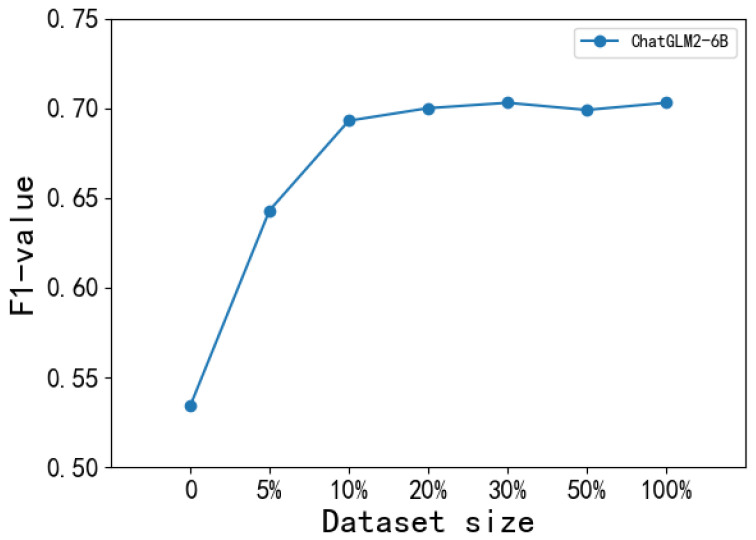
The performance of LLMs with the different sizes of the PCHD dataset.

**Figure 2:**
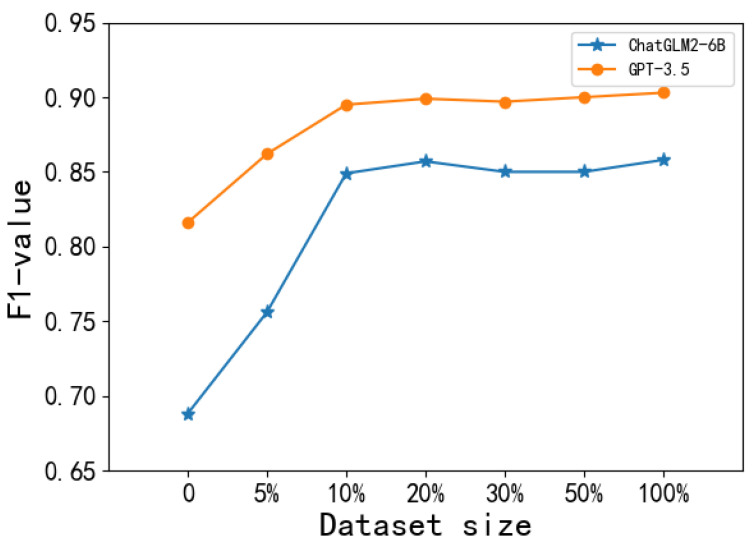
The performance of LLMs with the different sizes of the CCKS2017 dataset.

Specifically, we decrease the samples to 50%, 30%, 20%, 10%, and 5% of the original size of the fine-tuning datasets. Note that we conduct experiments based on LLMs with the best performance (i.e., ${\mathrm{ChatGLM2-6B}}_{PCHD\&CCKS}$ and ${\mathrm{GPT-3.5}}_{CCKS}$). The results are shown in Figure 1 and Figure 2.

We can observe from Figure 1 and Figure 2 that the performance of LLMs has an increasing trend with more fine-turned data, and this trend tends to stabilize after 10%. Furthermore, even when using only 10% of the original fine-tuning data, the LLMs already exhibit significant performance improvement. The difference in F1 values among 10% fine-tuning data and 100% fine-tuning data are only 0.009 and 0.008 on the PCHD dataset and the CCKS2017 dataset, respectively. This indicates that LLMs fine-tuning with a small number of labeled data can already obtain good performance.

The key points provided in the instruction fine-tuning section as follows:

(1) Similarly to few-shot, instruction fine-tuning appears to have a more pronounced effect on performance improvement for smaller-size LLMs.

(2) Under the same data conditions, fine-tuning LLMs with larger parameter sizes remain the primary choice for achieving high accuracy.

(3) While instruction fine-tuning has improved the performance of LLMs on the BNER task, there still remains a significant gap compared to state-of-the-art task-specific models.

(4) If the dataset scale is same, fine-tuning LLMs adopting the dataset with larger content changes can receive better performance.

(5) For specific datasets, fine-tuning LLMs with a small amount of labeled data from the dataset can already achieve excellent performance.

### 5.4. Resource Cost

As summarized in Section 5, models of a larger scale, such as GLM-130B, GPT-3.5, and GPT-4 demonstrate superior performance compared to the smaller-scale ChatGLM2-6B. However, larger scale typically implies an increasing demand for resource costs, particularly when it comes to instruction fine-tuning. In practical applications, beyond accuracy, a balanced consideration of computational costs, power consumption, and other multifaceted requirements is essential to determine the optimal LLM.

Table 9 provides an overview of the resource cost estimates for ChatGLM2-6B, GLM-130B, GPT-3.5, and GPT-4 on the CCKS dataset in this study (note that some of the LLMs used in this study, such as ChatGLM2-6B and GLM-130B, have been discontinued on the official websites (https://open.bigmodel.cn/ (accessed on 25 June 2023), https://openai.com/ (accessed on 14 March 2023)). Moreover, the official documents for different types of LLMs does not explicitly provide information on their respective energy consumption costs. Consequently, the data presented in the table is roughly estimated based on the actual costs of our experiments and some related reports on LLMs [53]). Reflecting on the performance of the LLMs, as indicated in Table 3, the following key points can be summarized.

(1) ChatGLM2-6B, with its smaller parameter scale, is clearly the best choice in scenarios where resource costs are highly constrained.

(2) If resources are average but not abundant, ChatGLM2-6B remains the optimal selection. On the one hand, ChatGLM2-6B has a low fine-tuning cost, and after fine-tuning, it achieves a high performance level (the accuracy of ${\mathrm{ChatGLM2-6B}}_{CCKS}$ has surpassed the basic GPT-4 and is only 0.043 behind the best ${\mathrm{GPT-3.5}}_{CCKS}$). On the other hand, being the smallest in parameter scale, ChatGLM2-6B also results in the least power consumption. In contrast, while GPT-3.5 has a good fine-tuning effect, its computational cost and power consumption are relatively high, preventing maximization of cost-effectiveness in scenarios with average resource conditions.

(3) For situations where resources are ample and high precision is a priority, the optimal model can be designed based on the availability of annotated data: in the absence of annotated data, the benefits of the larger parameter scale of GPT-4 are evident; if annotated data are available, GPT-3.5 demonstrates the highest cost-effectiveness in terms of both fine-tuning accuracy and resource expenditure.

## 6. Discussion

The experimental results in Section 5 summarized a number of our findings. In this section, we leverage these findings to discuss some guidelines for empowering LLMs for Chinese BNER tasks. Additionally, we also provide an analysis of privacy and bias issues related to LLMs, an explanation of the limitations of our current work, as well as directions and plans for future research endeavors.

### 6.1. Guidelines for Empowering LLMs for Chinese BNER Tasks

Based on the findings from Section 5, we offer some guidelines for future researchers on how to empower LLMs with stronger capability and become an expert for Chinese BNER tasks.

Design prompts carefully. The key points summarized in Section 5 indicate that the introduction of the enhancement strategies in zero-shot and few-shot prompt engineering is generally beneficial. Specifically, introducing the contextual information about the task can obtain significant improvements. Specifying the role of the model can also receive promising yet very limited help. The combination of multiple enhancement strategies usually confers the best performance gains, especially for LLMs with large trainable parameters.

Provide as many shots as possible. Having a greater number of shots aids the LLM in acquiring richer information, assisting the model in better understanding tasks, categories, or patterns, ultimately enhancing its accuracy and generalization capabilities.

Select an LLM with an appropriate parameter size based on specific requirements. The increase in the size of LLMs leads to an increase in the requirements for the computing power (mainly GPU), especially fine-tuning LLMs. As reflected in the results of Section 5, when faced with extremely limited conditional resources, a smaller-scale parameter LLM is undoubtedly the only viable option. Secondly, in situations where resources are average but not abundant, a smaller-scale parameter LLM offers the best balance in terms of precision, energy consumption, and computational cost. Particularly, setting aside the cost factor, if the goal is to achieve significant performance improvement or quickly adapt to new tasks in a short period, a smaller-scale parameter LLM also holds a distinct advantage. Lastly, for those with superior resource conditions seeking higher precision, the benefits of opting for an LLM with a larger parameter size will be more pronounced.

Prioritize selecting data with larger content changes. The experimental results shown in Section 5 indicate that when the scale of dataset is fixed, collecting the data with more content changes is more helpful, as the effects of instruction fine-tuning is better when the larger content variation.

Effective fine-tuning can be achieved with a small amount of data. As the results shown in Figure 1 and Figure 2, instruction fine-tuning does not require a large amount of data. Leveraging a small scale of data samples is generally sufficient when the data resource is limited.

### 6.2. Bias and Privacy

Although our experiments have demonstrated the exceptional capabilities of LLMs in medical information extraction tasks, there are several aspects that need careful attention and control when applying these models to clinical practice, such as bias and privacy.

Recent studies have uncovered potential biases and even harmful suggestions proposed by LLMs [54], particularly in terms of gender [55] and race [56]. Despite the dataset we used being meticulously annotated by human experts, there remains a risk of underlying biases in the labels, such as stereotypes [57] and confirmation biases [58]. Therefore, a significant focus and effort in our future work should be directed towards mitigating the bias issues in LLMs to promote the development of more equitable and just clinical application models and systems.

Moreover, privacy is a critical issue that demands careful consideration, especially in research involving private datasets. The private data leveraged in this study has undergone strict anonymization and de-identification procedures. These measures to protect patient privacy must be implemented and adhered to in any future research processes. To prevent and address potential ethical and moral risks, serious efforts are required from us and any other relevant parties in areas such as auditing, regulation, and secure development.

### 6.3. Limitations

The limitations of our study are obvious. Firstly, although we evaluated different categories of LLMs on the Chinese BNER tasks, the scale of the datasets and types of LLMs are still limited. Meanwhile, our findings are obtained from the observations of these LLMs and the domain-specific datasets, which may not be applicable to other cases. Further, prompt engineering is a complex, the zero-shot and few-shot prompt designs experimented in our study are not comprehensive. The enhancement strategies and questions only contain two to four designs.

### 6.4. Future Work Directions

Firstly, in our subsequent research efforts, we will broaden the scope of our study by delving into a more diverse array of datasets and a wider range of LLMs. The specific strategies involve introducing and integrating data resources that span across different professional fields, regions, and languages, with the goal of enhancing the generality of our research outcomes. Additionally, we plan to include more advanced LLMs, such as Llama [59,60], Spark Desk (demo: https://xinghuo.xfyun.cn/desk (accessed on 05 September 2023)), and Tongyi (demo: https://tongyi.aliyun.com/qianwen/ (accessed on 13 September 2023)) to deepen and expand the breadth of our research efforts. Through this comprehensive and in-depth exploration, we anticipate making more advancements in improving the domain adaptability and generalization capabilities of LLMs, thus compiling a more refined set of operational guidelines to offer unique insights for further research and application of LLMs in related fields.

Secondly, we will further enhance the design of prompt engineering in our future work. Specifically, we will systematically study the effects of various prompting strategies, such as Chain of Thought [61] and Step-by-Step Reasoning [62], to explore the boundaries of diverse prompting methods on model performance. Meanwhile, we will integrate external knowledge resources, e.g., knowledge bases and knowledge graphs, and design different knowledge prompting strategies to deeply validate and analyze the positive impact of knowledge on enhancing LLM performance.

Thirdly, promoting the transformation and sustainable development of research outcomes is also a key aspect of our future work. In this process, strengthening collaboration with hospitals is particularly important. Specifically, the transformation of research outcomes requires models with high precision, which necessitates diverse, large-scale, and high-quality annotated data. Medical experts can meet our data needs. Furthermore, promoting sustainable development typically requires models to incorporate domain-specific knowledge or new knowledge to enhance generalization capabilities. Medical experts, with their rich clinical experience and medical knowledge, can provide professional guidance and unique insights for model learning and reasoning, thereby enhancing the model’s generalizability.

Fourthly, while expanding the scope of our research, we will focus on addressing data bias and privacy issues to contribute to fairer, less biased, and privacy-preserving practical applications. The methods can be employed include: (1) anonymizing and de-identifying data during the data processing stage (i.e., removing or obfuscating information that identifies individuals in the data, such as gender, race, etc.); (2) adopting techniques like federated learning [63] and swarm learning [64] during the modeling process, which integrate local updates to form a global model, thus preventing sensitive data from leaving the original device; and (3) strengthening collaboration with domain-specific experts who much more understand of the importance and sensitivity of the data in their field, as well as privacy and security requirements. They can provide valuable insights on ensuring data privacy protection and compliance.

## 7. Conclusions

In this paper, we conduct the first extensive evaluations of multiple LLMs’ performance on Chinese BNER tasks. The evaluated experiments include zero-shot prompting, few-shot prompting, and instruction fine-tuning. Our results provide some useful information. The three proposed enhancement strategies can effectively improve the performance of LLMs, especially evident in models with larger trainable parameters. Meanwhile, few-shot prompting can also provide positive yet limited performance of LLMs. Impressively, we observed that instruction fine-tuning can significantly improve the LLMs’ performance on Chinese BNER tasks. The best fine-tuned models, ChatGLM2-6B and GPT-3.5, demonstrated outstanding performance on two distinct datasets. Notably, the fine-tuned model of ChatGLM2-6B surpassed the performance of a task-specific model BC (${\mathrm{ChatGLM2-6B}}_{PCHD}$ and ${\mathrm{ChatGLM2-6B}}_{PCHD\&CCKS}$ exceeded BC model by 0.036 and 0.045 on the PCHD dataset, while ${\mathrm{ChatGLM2-6B}}_{CCKS}$ and ${\mathrm{ChatGLM2-6B}}_{PCHD\&CCKS}$ surpassed BC model by 0.003 and 0.021 on the CCKS2017 dataset). Furthermore, fine-tuning ${\mathrm{GPT-3.5}}_{CCKS}$ on the CCKS2017 dataset outperformed more than half of the baseline models, i.e., BC, BERT, BBC, and RSBGC, with F1 values improving by 0.064, 0.038, 0.031, and 0.019, respectively. Finally, we summarize our research findings as a set of guidelines for future researchers and elaborate the future development directions to help LLMs better complete downstream tasks.

## Figures and Tables

**Table 1:** The definitions of *S_text* and *Q_text*. We set different versions for each part to explore the variations of results. Note that the examples are based on the PCHD dataset, and the entity categories vary across different datasets.

*S_text*	
Basic	{}
Context enhancement	(1) 这是一个命名实体识别任务, 注意实体的类别只有“症状、检查、检查结果、疾病、治疗”五种。(This is a named entity recognition task, note that there are only five categories of entities: ‘symptom’, ‘test’, ‘test result’, ‘disease’, and ‘treatment’.) (2) 这是一个命名实体识别任务, 注意只考虑“症状、检查、检查结果、疾病、治疗”这五种实体类别。(This is a named entity recognition task, note that only considering five entity categories: ‘symptom’, ‘test’, ‘test result’, ‘disease’, and ‘treatment’.)
Role-playing	(1) 作为一名医学专家, 请阅读这条电子病历文本并回答这个问题。(As a medical expert, please read this electronic medical record text and answer this question.) (2) 如果你是一名医学专家, 请阅读这条电子病历文本并回答这个问题。(If you are a medical expert, please read this electronic medical record text and answer this question.)
Context and Role-playing	(1) 这是一个命名实体识别任务, 注意实体类别只有“症状、检查、检查结果、疾病、治疗”五种。作为一名医学专家, 请阅读这条电子病历文本并回答这个问题。(This is a named entity recognition task, note that there are only five categories of entities: ‘symptom’, ‘test’, ‘test result’, ‘disease’, and ‘treatment’. As a medical expert, please read this electronic medical record text and answer this question.) (2) 这是一个命名实体识别任务, 注意只考虑“症状、检查、检查结果、疾病、治疗”这五种实体类别。作为一名医学专家, 请阅读这条电子病历文本并回答这个问题。(This is a named entity recognition task, note that only considering five entity categories: ‘symptom’, ‘test’, ‘test result’, ‘disease’, and ‘treatment’. As a medical expert, please read this electronic medical record text and answer this question.) (3) 这是一个命名实体识别任务, 注意实体类别只有“症状、检查、检查结果、疾病、治疗”五种。如果你是一名医学专家, 请阅读这条电子病历文本并回答这个问题。(This is a named entity recognition task, note that there are only five categories of entities: ‘symptom’, ‘test’, ‘test result’, ‘disease’, and ‘treatment’. If you are a medical expert, please read this electronic medical record text and answer this question.) (4) 这是一个命名实体识别任务, 注意只考虑“症状、检查、检查结果、疾病、治疗”这五种实体类别。如果你是一名医学专家, 请阅读这条电子病历文本并回答这个问题。(This is a named entity recognition task, note that only considering five entity categories: ‘symptom’, ‘test’, ‘test result’, ‘disease’, and ‘treatment’. If you are a medical expert, please read this electronic medical record text and answer this question.)
***Q_text***	
Entity recognition and type prediction	(1) 请标记出这条文本中的医学实体, 并相应的给出这些实体所属的类型。(Please label the biomedical entities in this text and provide their corresponding types.) (2) 这条文本中的医学实体有哪些, 请识别出这些实体并给出其所属的类型。(What are the biomedical entities in this text? Please recognize these entities and provide their corresponding types.)

**Table 2:** Hardware configuration.

Processor	Cache	GPU	Hard Disk
13th Gen Intel Core i9-13900KF × 32	128 GB	NVIDIA Corporation 4090 24 GB	3.0 TB

**Table 3:** The experiment results on the PCHD dataset.

Type	Category	Models	P	R	F1
Supervised	Baseline	BC [24]	0.681 ± 0.001	0.670 ± 0.002	0.675 ± 0.001
		BERT [49]	0.744 ± 0.017	0.721 ± 0.010	0.732 ± 0.015
		BBC [50]	0.748 ± 0.008	0.732 ± 0.015	0.740 ± 0.012
		RSBGC [51]	0.756 ± 0.006	0.763± 0.010	0.760 ± 0.008
		FBBCE [52]	0.802 ± 0.005	0.803 ± 0.010	0.802 ± 0.007
		DABLC [27]	0.815 ± 0.004	0.812 ± 0.004	0.813 ± 0.003
		DGAN [28]	0.832 ± 0.000	0.820 ± 0.000	0.826 ± 0.000
LLMs	Zero-shot	${\mathrm{ChatGLM2-6B}}_{basic}$	0.388 ± 0.023	0.402 ± 0.015	0.395 ± 0.020
		${\mathrm{ChatGLM2-6B}}_{context}$	0.500 ± 0.031	0.592 ± 0.041	0.542 ± 0.036
		${\mathrm{ChatGLM2-6B}}_{rp}$	0.482 ± 0.010	0.501 ± 0.039	0.491 ± 0.022
		${\mathrm{ChatGLM2-6B}}_{both}$	0.551 ± 0.031	0.567 ± 0.020	0.559 ± 0.025
LLMs	Few-shot	${\mathrm{ChatGLM2-6B}}_{5-shot}$	0.600 ± 0.014	0.588 ± 0.019	0.594 ± 0.018
		${\mathrm{ChatGLM2-6B}}_{10-shot}$	0.613 ± 0.017	0.609 ± 0.010	0.611 ± 0.012
LLMs	Fine-tuning	${\mathrm{ChatGLM2-6B}}_{PCHD}$	0.694 ± 0.032	0.730 ± 0.023	0.711 ± 0.025
		${\mathrm{ChatGLM2-6B}}_{CCKS}$	0.576 ± 0.021	0.590 ± 0.018	0.583 ± 0.018
		${\mathrm{ChatGLM2-6B}}_{PCHD\&CCKS}$	0.720 ± 0.010	0.721 ± 0.017	0.720 ± 0.012

**Table 4:** The experiment results on the CCKS2017 dataset.

Type	Category	Models	P	R	F1
Supervised	Baseline	BC [24]	0.836 ± 0.001	0.849 ± 0.001	0.842 ± 0.001
		BERT [49]	0.879 ± 0.003	0.858 ± 0.007	0.868 ± 0.004
		BBC [50]	0.877 ± 0.009	0.873 ± 0.010	0.875 ± 0.007
		RSBGC [51]	0.891 ± 0.004	0.884 ± 0.003	0.887 ± 0.004
		FBBCE [52]	0.920 ± 0.003	0.913 ± 0.002	0.916 ± 0.003
		DABLC [27]	0.925 ± 0.002	0.919 ± 0.001	0.922 ± 0.001
		DGAN [28]	0.950 ± 0.001	0.954 ± 0.001	0.952 ± 0.001
LLMs	Zero-shot	${\mathrm{ChatGLM2-6B}}_{basic}$	0.546 ± 0.024	0.600 ± 0.037	0.572 ± 0.030
		${\mathrm{ChatGLM2-6B}}_{context}$	0.615 ± 0.041	0.652 ± 0.038	0.633 ± 0.040
		${\mathrm{ChatGLM2-6B}}_{rp}$	0.600 ± 0.031	0.567 ± 0.043	0.583 ± 0.037
		${\mathrm{ChatGLM2-6B}}_{both}$	0.672 ± 0.056	0.641 ± 0.022	0.648 ± 0.039
		${\mathrm{GLM-130B}}_{basic}$	0.642 ± 0.004	0.599 ± 0.019	0.620 ± 0.013
		${\mathrm{GLM-130B}}_{context}$	0.771 ± 0.030	0.723 ± 0.011	0.746 ± 0.020
		${\mathrm{GLM-130B}}_{rp}$	0.648 ± 0.022	0.627 ± 0.039	0.637 ± 0.033
		${\mathrm{GLM-130B}}_{both}$	0.748 ± 0.005	0.804 ± 0.044	0.775 ± 0.039
		${\mathrm{GPT-3.5}}_{basic}$	0.663 ± 0.041	0.621 ± 0.044	0.641 ± 0.045
		${\mathrm{GPT-3.5}}_{context}$	0.788 ± 0.009	0.740 ± 0.014	0.763 ± 0.011
		${\mathrm{GPT-3.5}}_{rp}$	0.732 ± 0.066	0.677 ± 0.017	0.703 ± 0.049
		${\mathrm{GPT-3.5}}_{both}$	0.802 ± 0.022	0.776 ± 0.030	0.789 ± 0.025
		${\mathrm{GPT-4}}_{basic}$	0.704 ± 0.077	0.660 ± 0.064	0.681 ± 0.065
		${\mathrm{GPT-4}}_{context}$	0.732 ± 0.036	0.819 ± 0.038	0.773 ± 0.038
		${\mathrm{GPT-4}}_{rp}$	0.750 ± 0.017	0.711 ± 0.015	0.730 ± 0.016
		${\mathrm{GPT-4}}_{both}$	0.784 ± 0.031	0.801 ± 0.009	0.792 ± 0.019
LLMs	Few-shot	${\mathrm{ChatGLM2-6B}}_{5-shot}$	0.660 ± 0.035	0.710 ± 0.024	0.684 ± 0.030
		${\mathrm{GLM-130B}}_{5-shot}$	0.799 ± 0.010	0.773 ± 0.017	0.786 ± 0.015
		${\mathrm{GPT-3.5}}_{5-shot}$	0.820 ± 0.027	0.802 ± 0.014	0.811 ± 0.021
		${\mathrm{GPT-4}}_{5-shot}$	0.813 ± 0.011	0.832 ± 0.005	0.822 ± 0.007
		${\mathrm{ChatGLM2-6B}}_{10-shot}$	0.675 ± 0.047	0.702 ± 0.056	0.688 ± 0.060
		${\mathrm{GLM-130B}}_{10-shot}$	0.818 ± 0.009	0.800 ± 0.032	0.809 ± 0.020
		${\mathrm{GPT-3.5}}_{10-shot}$	0.837 ± 0.016	0.796 ± 0.020	0.816 ± 0.018
		${\mathrm{GPT-4}}_{10-shot}$	0.822 ± 0.006	0.838 ± 0.008	0.830 ± 0.006
LLMs	Fine-tuning	${\mathrm{ChatGLM2-6B}}_{PCHD}$	0.759 ± 0.023	0.702 ± 0.018	0.729 ± 0.021
		${\mathrm{ChatGLM2-6B}}_{CCKS}$	0.830 ± 0.006	0.861 ± 0.004	0.845 ± 0.005
		${\mathrm{ChatGLM2-6B}}_{PCHD\&CCKS}$	0.867 ± 0.012	0.860 ± 0.009	0.863 ± 0.010
		${\mathrm{GPT-3.5}}_{CCKS}$	0.897 ± 0.009	0.915 ± 0.003	0.906 ± 0.005

**Table 5:** The performance changes of LLMs using the enhancement strategies of zero-shot prompting.

Models	PCHD			CCKS2017		
	P	R	F1	P	R	F1
${\mathrm{ChatGLM2-6B}}_{context}$	↑0.112	↑0.190	↑0.147	↑0.069	↑0.052	↑0.061
${\mathrm{ChatGLM2-6B}}_{rp}$	↑0.094	↑0.099	↑0.096	↑0.054	↓0.033	↑0.011
${\mathrm{ChatGLM2-6B}}_{both}$	↑0.163	↑0.165	↑0.164	↑0.126	↑0.041	↑0.076
${\mathrm{GLM-130B}}_{context}$	-	-	-	↑0.129	↑0.124	↑0.126
${\mathrm{GLM-130B}}_{rp}$	-	-	-	↑0.006	↑0.028	↑0.017
${\mathrm{GLM-130B}}_{both}$	-	-	-	↑0.105	↑0.205	↑0.155
${\mathrm{GPT-3.5}}_{context}$	-	-	-	↑0.125	↑0.119	↑0.122
${\mathrm{GPT-3.5}}_{rp}$	-	-	-	↑0.069	↑0.056	↑0.062
${\mathrm{GPT-3.5}}_{both}$	-	-	-	↑0.139	↑0.155	↑0.148
${\mathrm{GPT-4}}_{context}$	-	-	-	↑0.028	↑0.159	↑0.092
${\mathrm{GPT-4}}_{rp}$	-	-	-	↑0.046	↑0.051	↑0.049
${\mathrm{GPT-4}}_{both}$	-	-	-	↑0.080	↑0.141	↑0.111

**Table 6:** The performance changes of LLMs using the enhancement strategies of few-shot prompting.

Models	PCHD			CCKS2017		
	P	R	F1	P	R	F1
${\mathrm{ChatGLM2-6B}}_{5-shot}$	↑0.049	↓0.021	↑0.035	↓0.012	↑0.069	↑0.036
${\mathrm{GLM-130B}}_{5-shot}$	-	-	-	↑0.051	↓0.031	↑0.011
${\mathrm{GPT-3.5}}_{5-shot}$	-	-	-	↑0.018	↑0.026	↑0.021
${\mathrm{GPT-4}}_{5-shot}$	-	-	-	↑0.029	↑0.031	↑0.030
${\mathrm{ChatGLM2-6B}}_{10-shot}$	↑0.062	↑0.042	↑0.052	↑0.003	↑0.061	↑0.040
${\mathrm{GLM-130B}}_{10-shot}$	-	-	-	↑0.070	↓0.004	↑0.034
${\mathrm{GPT-3.5}}_{10-shot}$	-	-	-	↑0.035	↑0.020	↑0.027
${\mathrm{GPT-4}}_{10-shot}$	-	-	-	↑0.038	↑0.037	↑0.038

**Table 7:** The performance changes of LLMs with instruction fine-tuning.

Models	PCHD			CCKS2017		
	P	R	F1	P	R	F1
${\mathrm{ChatGLM2-6B}}_{PCHD}$	↑0.143	↑0.163	↑0.152	↑0.087	↑0.061	↑0.081
${\mathrm{ChatGLM2-6B}}_{CCKS}$	↑0.025	↓0.023	↑0.024	↑0.158	↑0.220	↑0.197
${\mathrm{ChatGLM2-6B}}_{PCHD\&CCKS}$	↑0.169	↑0.154	↑0.161	↑0.195	↑0.219	↑0.215
${\mathrm{GPT-3.5}}_{CCKS}$	-	-	-	↑0.113	↑0.114	↑0.114

**Table 8:** Comparative experiments of fine-tuned LLMs and baselines.

Type	Models	PCHD			CCKS2017		
		P	R	F1	P	R	F1
Baseline	BC [24]	0.836 ± 0.001	0.849 ± 0.001	0.842 ± 0.001	0.836 ± 0.001	0.849 ± 0.001	0.842 ± 0.001
	BERT [49]	0.879 ± 0.003	0.858 ± 0.007	0.868 ± 0.004	0.879 ± 0.003	0.858 ± 0.007	0.868 ± 0.004
	BBC [50]	0.877 ± 0.009	0.873 ± 0.010	0.875 ± 0.007	0.877 ± 0.009	0.873 ± 0.010	0.875 ± 0.007
	RSBGC [51]	0.891 ± 0.004	0.884 ± 0.003	0.887 ± 0.004	0.891 ± 0.004	0.884 ± 0.003	0.887 ± 0.004
	FBBCE [52]	0.920 ± 0.003	0.913 ± 0.002	0.916 ± 0.003	0.920 ± 0.003	0.913 ± 0.002	0.916 ± 0.003
	DABLC [27]	0.925 ± 0.002	0.919 ± 0.001	0.922 ± 0.001	0.925 ± 0.002	0.919 ± 0.001	0.922 ± 0.001
	DGAN [28]	0.950 ± 0.001	0.954 ± 0.001	0.952 ± 0.001	0.950 ± 0.001	0.954 ± 0.001	0.952 ± 0.001
Fine-tuned LLM	${\mathrm{ChatGLM2-6B}}_{PCHD}$	0.694 ± 0.032	0.730 ± 0.023	0.711 ± 0.025	0.759 ± 0.023	0.702 ± 0.018	0.729 ± 0.021
	${\mathrm{ChatGLM2-6B}}_{CCKS}$	0.576 ± 0.021	0.590 ± 0.018	0.583 ± 0.018	0.830 ± 0.006	0.861 ± 0.004	0.845 ± 0.005
	${\mathrm{ChatGLM2-6B}}_{PCHD\&CCKS}$	0.720 ± 0.010	0.721 ± 0.017	0.720 ± 0.012	0.867 ± 0.012	0.860 ± 0.009	0.863 ± 0.010
	${\mathrm{GPT-3.5}}_{CCKS}$	-	-	-	0.897 ± 0.009	0.915 ± 0.003	0.906 ± 0.005

**Table 9:** The overview of resource costs for different LLMs.

Models	CCKS2017	
	Computational Costs ($)	Power Consumption (kWh)
ChatGLM2-6B	3.93	52.10
GLM-130B	12.45	1092.45
GPT-3.5	14.32	1470.60
GPT-4	58.79	15,125.79

## Data Availability

The data presented in this study are available on request from the corresponding author due to privacy, legal or ethical reasons.
